# A Serious Game to Train Rhythmic Abilities in Children With Dyslexia: Feasibility and Usability Study

**DOI:** 10.2196/42733

**Published:** 2024-01-11

**Authors:** Francois Vonthron, Antoine Yuen, Hugues Pellerin, David Cohen, Charline Grossard

**Affiliations:** 1 Poppins Palaiseau France; 2 Service de Psychiatrie de l'Enfant et de l'Adolescent, Groupe Hospitalier Pitié-Salpêtrière, Assistance Publique–Hôpitaux de Paris Paris France; 3 Institut des Systèmes Intelligents et Robotiques (ISIR, CNRS UMR7222), Sorbonne Université Paris France

**Keywords:** serious game, rhythm, dyslexia, musical abilities, design framework, reading skills, children, digital health intervention, attention-deficit/hyperactivity disorder, ADHD, child development, mobile phone

## Abstract

**Background:**

Rhythm perception and production are related to phonological awareness and reading performance, and rhythmic deficits have been reported in dyslexia. In addition, rhythm-based interventions can improve cognitive function, and there is consistent evidence suggesting that they are an efficient tool for training reading skills in dyslexia.

**Objective:**

This paper describes a rhythmic training protocol for children with dyslexia provided through a serious game (SG) called Mila-Learn and the methodology used to test its usability.

**Methods:**

We computed Mila-Learn, an SG that makes training remotely accessible and consistently reproducible and follows an educative agenda using Unity (Unity Technologies). The SG’s development was informed by 2 studies conducted during the French COVID-19 lockdowns. Study 1 was a feasibility study evaluating the autonomous use of Mila-Learn with 2500 children with reading deficits. Data were analyzed from a subsample of 525 children who spontaneously played at least 15 (median 42) games. Study 2, following the same real-life setting as study 1, evaluated the usability of an enhanced version of Mila-Learn over 6 months in a sample of 3337 children. The analysis was carried out in 98 children with available diagnoses.

**Results:**

Benefiting from study 1 feedback, we improved Mila-Learn to enhance motivation and learning by adding specific features, including customization, storylines, humor, and increasing difficulty. Linear mixed models showed that performance improved over time. The scores were better for older children (*P*<.001), children with attention-deficit/hyperactivity disorder (*P*<.001), and children with dyslexia (*P*<.001). Performance improved significantly faster in children with attention-deficit/hyperactivity disorder (β=.06; *t*_3754_=3.91; *P*<.001) and slower in children with dyslexia (β=−.06; *t*_3816_=–5.08; *P*<.001).

**Conclusions:**

Given these encouraging results, future work will focus on the clinical evaluation of Mila-Learn through a large double-blind randomized controlled trial comparing Mila-Learn and a placebo game.

## Introduction

### Background

Music training and music-based interventions are becoming increasingly popular for developing brain and cognitive functions in children [1-5]. Building on brain plasticity induced by learning music and the tight link between musical and cognitive skills [6-8], music interventions have been used as training tools in neurodevelopmental disorders (NDDs) such as dyslexia [9-13]. Musical skills, especially when developed in childhood, are associated with enhanced cognitive abilities in various domains, such as attention, processing speed [3], executive functions [14], or speech and language [15-17]. Improvements in cognitive skills induced by musical training have been attributed to structural and functional brain changes in areas that support both music processing and cognition [6-8,18,19].

Recent studies have focused specifically on the relationship between rhythmic skills, such as the capacity to discriminate musical rhythms or synchronize with a beat [20,21], and cognition during development. Tierney and Kraus [22] showed that correlations exist between synchronization with a metronome and attentional and reading skills in typically developing adolescents. In children, rhythm production accuracy is associated with both phonological awareness and reading [23]. Rhythm perception is also related to reading performance [24,25]. Language and music processing may rely on common timing mechanisms that allow for the extraction of temporal information, which is crucial to accurately perceive sequences of events [7,20,26-28]. This hypothesis is supported by neurofunctional evidence as temporal processing involved in music and language recruits partially overlapping neuronal pathways that include the auditory cortex, dorsal premotor cortex, cerebellum, basal ganglia, and thalamus [29,30].

Further evidence of the link between rhythmic skills and cognitive abilities comes from the observation that rhythmic skills are disrupted in NDDs that also affect cognition. Notably, rhythmic deficits have been extensively reported in individuals with dyslexia. Children and adults with dyslexia exhibit inaccurate rhythm perception [25,31] as well as increased variability in motor tapping tasks [32]. These observed rhythmic deficits have given rise to theories (eg, the temporal sampling framework; Goswami [26]) that postulate that poor predictive temporal sampling and coding of events explain reading difficulties in those with dyslexia [26,33].

Building on the importance of rhythmic skills in development, music-based training protocols for children have been developed in recent decades. Studies have shown that children with dyslexia who participate in music-based interventions display better reading and phonological abilities [10-12]. In addition, the effect of music-based programs was extended to typically developing children, who showed significant improvements in speech processing skills and verbal intelligence [14]. However, these encouraging preliminary data have not reached the recommended quality for evidence-based studies owing to methodological limitations such as limited sample size, lack of blind assessment, and potentially inconsistent delivery of interventions [34]. In addition, access to these interventions is still too limited, with inequalities remaining because of significant disparities according to social background and place of residence [35]. For instance, children in poor and remote urban areas, who are more likely to develop an NDD [36], often have less access to care. Furthermore, these traditional music-based interventions usually require in-person instruction, which can be challenging under certain circumstances such as during the COVID-19 pandemic or in areas with limited access to specialized resources. More research is needed to determine whether written language skills can improve in children with dyslexia after training with more accessible and scalable music-based interventions.

To address these limitations, serious games (SGs) designed for educational and training purposes provide a more standardized, scalable, and accessible format for delivering music-based interventions through information and communications technologies. This approach allows for the delivery of the same training to a large sample regardless of geographic location or in-person resource availability. The number of SGs developed for educational and training purposes has increased over the last decade [37], primarily because of the expansion of information and communications technologies such as mobile technologies and telehealth systems. As most households, including those in low-income brackets and rural areas, are now equipped with at least 1 tablet, smartphone, or computer, these SGs can be broadly accessible [35]. Furthermore, a meta-analysis revealed that, across domains, learning is improved with SGs compared with conventional methods [38]. In addition to motivation, several preliminary findings have supported another exciting alternative hypothesis that playing an SG fosters electrical brain activity related to memory, emotions, and concentration [39], providing a possible neuronal explanation for the beneficial effect of SGs. SGs have been used in typically developing populations [40] and in children with NDDs [41,42]. Notably, SGs have been used to deliver rhythm-based training to healthy young adults [43]. Recently, interest in using computer-based interventions to train rhythm skills has been explored in people with dyslexia [44]. One SG named “Jellys” was developed for this purpose in a usability study and showed that children with dyslexia positively engaged with this type of remediation [45]. However, although some studies seem to support the effectiveness of using SGs as a treatment for people with NDDs, the methodological quality of these studies is limited, and further research is needed [46].

### Objectives

In this study, our goal was to evaluate the usability of Mila-Learn, an SG aimed at training rhythmic abilities in children with dyslexia. The methodological design of the game was developed in user participatory pilot studies, allowing the children and their families to provide feedback to shape human-machine interactions. We report on 2 studies conducted during the French COVID-19 lockdowns. The first was a feasibility study to assess the children’s engagement through gameplay frequency and collected their feedback. After modifying the SG according to the feedback by adding specific features such as customization, storyline, humor, or increasing difficulty, we report a usability study that addressed the children’s performance on the latest version of the SG when played autonomously at home and according to declared diagnoses.

## Methods

### Overview

Mila-Learn is an SG that delivers rhythm-based exercise designed for children with dyslexia (called “specific learning disorders in the field of reading” in the *Diagnostic and Statistical Manual of Mental Disorders, Fifth Edition*). This SG involves a rich musical universe aiming to lead the child to spontaneously come back and engage with the instrument with their parents. It consists of two main elements: (1) a mobile app that offers rhythmic, sensory-motor, and cognitive tasks in the form of musical activities; and (2) secure servers that allow for data analysis. It enables real-time evaluations to understand children’s difficulties and improve the effectiveness of Mila-Learn. In this section, we first describe the SG from its initial beta version. We then detail the methodology of the 2 exploratory studies conducted during the COVID-19 lockdown.

### Mila-Learn Description

#### From an Initial Prototype (2018) as a Progressive Web Application to a Version (2019-2020) Developed for Tablets

The first version of Mila-Learn included five tasks:

1. Dance With Your Hands is an auditory-motor coordination exercise. It involves performing a movement following the tempo of a piece of music. These are pieces with a 4/4 signature that easily allow the child to have rhythmic stability and associate a motor action with the rhythm. This action includes gestures such as clapping, silencing, and raising the arms in the air.
2. Play the Drums is a rhythmic memory game. A drum appears on the child’s screen, and a sequence is played. The child then presses on the drum elements to reproduce the initially played sequence.
3. Rhythmic Vitamins is an exercise in singing and repetition [47]. An initial recorded vocal sequence consisting of syllables and phonemes is played by the software. The child must reproduce it using the rhythm, pronunciation, and pitch of the initial sequence.
4. Following the Tempo requires recognizing and reproducing different rhythmic structures. In this exercise, the child is asked to mark the strong beats of music using the space key on the keyboard.
5. Musical Pitch is an exercise of association between the pitch and its representation. A sound sequence composed of 3 sounds is played (high, medium, and low), and then a visual representation is displayed composed of lines (low, medium, and high). The child has to judge whether the graphic production of the sound is correct.

**Figure 1:**
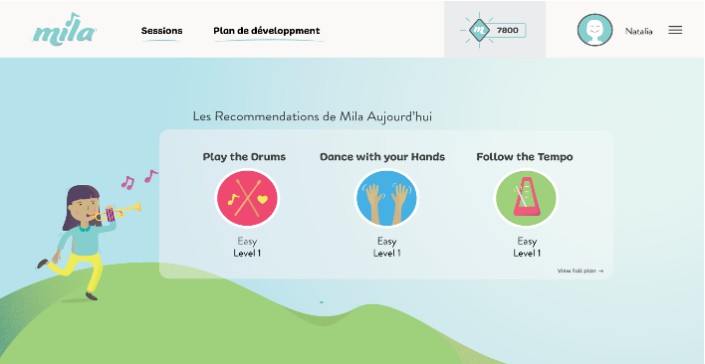
Landing page of the prototype with 3 different tasks each day [56].

This prototype version (Figure 1 [48]) was offered to a small group of children end users with dyslexia. We asked them to provide feedback on the design and players. The data collection method was centered on gathering children’s feedback at the end of 15-minute game sessions. A total of 14 children were invited to respond, interact, and provide feedback on the first version of Mila-Learn in the form of a progressive web application [49]. In total, 3 sessions per week over a period of 3 months were conducted.

This participatory design phase led to a framework for the development of Mila-Learn enhanced by a literature review on SG playability to increase players’ motivation. Although some studies have proposed a framework to develop SGs for people with NDDs, very few have focused specifically on dyslexia [46]. To improve the design of our game, we expanded our research to the use of SGs in the typical population and in children with NDDs, especially children with attention-deficit/hyperactivity disorder (ADHD), because of the high co-occurrence between dyslexia and ADHD [50].

#### Design Framework

We integrated new gaming features to increase players’ motivation and engagement. First, we developed a storyline that allowed us to include the different tasks within a larger story. The use of a storyline supported the engagement of a player in the games [51,52]. The storyline was intended to not be complex to prevent the child from losing the main goal of the game [53]. The story in Mila-Learn was designed to correspond to the interests of children aged between 7 and 14 years. The story is inspired by *shonen* manga, which is manga inspired by the cartoon universe. This type of manga is based on a storyline that involves a young hero who starts without knowledge and becomes increasingly powerful during the progression of the story. Some crucial values such as friendship and perseverance are typically present in the story. Most of the time, the first opponent of the hero becomes his friend during the story. In Mila-Learn, the player embodies a little monster who meets another character, a little blue monster named “Blue” who asks for their help—some villagers are held captive by the villain Diabolus, another character. The player has to learn rhythm skills to challenge Diabolus and free the villagers. Over the course of the game, the player discovers that there is a larger villain who holds “Rosa,” the Diabolus’ scooter. At the end, the player must win a large rhythm tournament to finish the game. The story is divided into 12 chapters containing 6 tasks (see the following section). Each task lasts between 1 minute, 20 seconds and 1 minute, 30 seconds, with rare songs playing for 1 minute, 40 seconds to maintain consistency with the music. In this way, we considered the attention capacity of children [53,54].

As recommended in the literature, we created evolving tasks, gradually increasing the level of difficulty in each task and from one task to another [51,53,54]. The tasks must be challenging but accessible. In total, 6 tasks are used in the second version of Mila-Learn. They are introduced progressively to allow the player to practice a task 2 to 3 times before introducing another one. Once all the tasks are known by the player, they increase in difficulty with progression throughout the game. First, within each task, the rhythm displayed corresponds to each beat of a measure. Then, the rhythm changes to correspond to eighth notes (meaning that the rhythm is clapping 3 times in 2 beats) or slows down to be marked only once every 2 beats. Moreover, the songs are played at an increased speed to challenge the player. At higher levels, the marked rhythms can change during the task.

The SG was built to provide clear instructions to the player [51,53]. The instructions are given orally and with visual support, notably by imitating one or more nonplayer characters. Before each task, a quick tutorial allows the player to repeat the movement they have to perform during the game 3 times (ie, clap their hands, touch the screen, and move the tablet). At the beginning of a task, the character played by the user is clearly identified with an arrow. Moreover, for each task, the player is always placed in the same location.

The visual environment is thought to be easily navigated by children. The graphics are pleasant but minimalistic [53,55]. The visuals are thought to be pleasant for children aged between 7 and 14 years and are inspired by the cartoon universe. During the tasks, the background is mostly static, allowing the child to focus on the goal of the task. The characters only move to the rhythm of the music, with repetitive and predictable movements.

**Figure 2:**
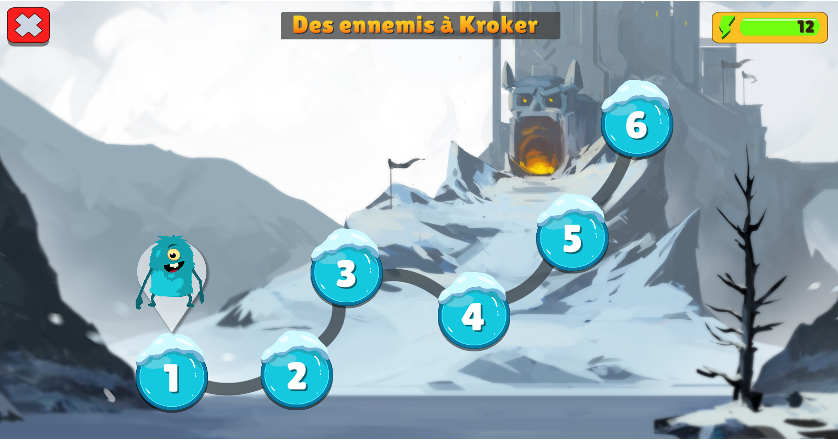
Example of a chapter menu introducing the upcoming adventure.

We differentiate between short- and long-term goals [51,53,54]. In each task, there is only 1 clear goal (ie, touch the screen to the rhythm) that is clearly differentiated from the long-term goal of a chapter (ie, complete the chapter to challenge Diabolus; Figure 2). Feedback is provided throughout the different tasks using visual cues [51,53,54]. These cues allow the player to know whether they are performing the exercise properly. The feedback for each task is described in the following section. As rewards have been described as a main feature of SGs [51,54], players obtain a reward of 1 to 3 stars at the end of each task depending on their accuracy during the exercise. Personalization has also been described as an important key to enhancing the motivation of the player [51-54]. As in the first version, players have to pick a name for their character at the beginning of the game and modify its color. Finally, we introduced new songs to work on in this version of the game. We added some famous songs known by most children (ie, songs from Disney movies) to increase the motivation of the players. For some tasks such as Fruity Jump, Karate Fruit, and Sing Lab, the predetermined structures of these songs did not make their use possible. We specifically composed songs to fit with the requirements of these tasks.

#### Description of the Tasks

**Figure 3:**
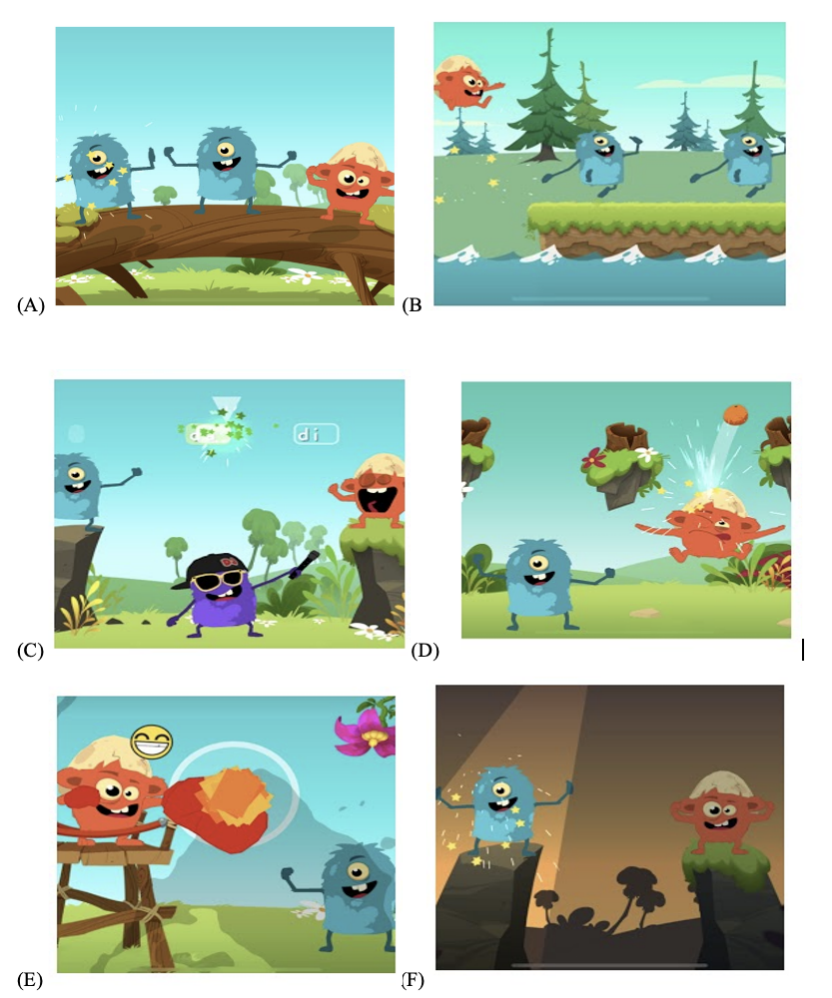
Examples of screens in Mila-Learn: (A) Clap Trap, (B) River Splash, (C) Sing Lab, (D) Fruity Jumps, (E) Karate Fruits, and (F) Follow Me.

All the tasks (Figure 3) were designed to work on rhythm, which was the main and explicit goal of each task. However, each task requires the mobilization of other skills such as attention, inhibition, working memory, and motor skills, which are also often impaired in children with dyslexia [50,56].

Follow me aims to introduce rhythm to the player. The child first sees a little monster clapping hands to the rhythm of a song and then has to touch the screen to the rhythm by imitating the monster. Then, the character stops clapping, and the player has to keep going alone without the support of the monster. This task allows the player to work on maintaining regularity in rhythm but also sustained attention.

In Clap Trap, 2 characters and the player appear on the screen. The first 2 characters clap one after another, giving a tempo to the player, who has to complete the sequence by clapping their hands to the rhythm at the right time. The first character claps on the first beat of a 4-time measure of the song played. The second character claps on the second beat, and the player has to clap their hands on the third beat. The microphone records the child’s clap. In this task, the child has to anticipate and adapt to the rhythm. It was designed to train inhibition skills as the child has to wait until the right moment to clap their hands.

In River Splash, the player is placed behind 2 other characters who run next to the water and sometimes have to jump across the river to the rhythm. The first character jumps on the first beat of a 4-time measure of a song. The second character jumps on the second beat, and the player jumps on the third beat. The player has to shake the tablet quickly to jump. In addition to rhythm perception, this task was designed to train inhibition skills similarly to the Clap Trap task.

In Sing Lab, the first character produces a sequence of phonemes or syllables at a particular tempo. The player has to reproduce this sequence with particular attention to the pattern and duration of the phonemes. Phonemes or syllables pass across the top of the screen, visually represented by gauges that the player has to fill. If the child sings at the right time, the gauge starts filling. When the duration of the note is complete, the gauge changes color from white to green. In this task, the phonological loop is involved in correctly memorizing the sequence. We used specific music constructed for this task that allowed us to add phonemes or syllables to sing at particular moments and for as long as we wanted.

In Fruity Jump, a character reproduces a rhythmic sequence. The player has to memorize this sequence and then reproduce it correctly by tapping the screen at a good tempo. The tempo is visually indicated by fruits falling from a tree. If the player claps at the right time, the character jumps and hits a fruit with its head to throw it to another tree. If the player misses the fruit, it crashes on the ground. If the player jumps at another time (ie, during the demonstration), their character jumps, and nothing special happens. Working memory and intermodality are particularly engaged in this task.

Finally, in Karate Fruits, the player has to hit fruits that appear based on the rhythm. To hit the fruit, the player has to put the tablet on the floor and extend their arms above it. Each time the camera detects the arm, the player’s character punches. If the player punches at the right time, the fruit explodes, and a smiley face appears. If the player misses the fruit, the fruit goes off the screen, and a smiley face with an annoyed head appears. If the player punches at another time, the character punches, and nothing else happens.

#### Scoring Player Performance

Scoring of player performance is based on rhythmic synchronization through multiple modalities of interaction (sometimes in combination) as rhythmic synchronization is a requirement for all games. Player responses are captured through accelerometers, microphones, webcams, and pressure-sensitive screens, as shown in Table 1.

By assessing the audiomotor synchronization of the child with the rhythmic instruction, we define (1) a time T that corresponds to the exact moment when the player’s input is expected (regardless of the interaction mode) and (2) tolerance thresholds (t_Perfect_<t_Good_<t_Correct_).

The different intervals allow for judging the quality of the answer with 4 levels of acceptance. An input is considered acceptable when it is in the interval [T – t_Correct_; T + t_Correct_] and not acceptable otherwise. An input of better quality, either in the interval [T – t_Good_; T + t_Good_] or in the interval [T – t_Perfect_; T + t_Perfect_], results in different visual and audio feedback for the child.

In the second improved version, which was a modified version based on the first pilot study, a simplified calculation was performed by considering the ratio of acceptable inputs to total inputs as the main measure. This final score is presented to the child in the form of stars depending on their performance: no stars if the child has an average of <50%, 1 star if ≥50% of inputs are acceptable, 2 stars for ≥75%, and 3 stars for ≥90%. In addition, this architecture allows for the storage of all the child’s inputs for retro-analysis purposes.

**Table 1:** Players’ recorded responses and game parameters in the second version of Mila-Learn.

Task	Type	Interaction	Capture technology	Songs	Tolerance threshold
Follow Me^a^	Continuous tapping	Tapping	Contact pressure	Commercial	t_Perfect_: 0.1 s before or after the beat t_Good_: 0.15 s before or after the beat t_Correct_: 0.25 s before or after the beat
Clap Trap	Last beat	Clapping hands	Microphone	Commercial	t_Perfect_: 0.1 s before or after the beat t_Good_: 0.15 s before or after the beat t_Correct_: 0.25 s before or after the beat
River Splash	Last beat	Shaking tablet	Accelerometer	Commercial	t_Perfect_: 0.1 s before or after the beat t_Good_: 0.15 s before or after the beat t_Correct_: 0.25 s before or after the beat
Sing Lab	Call and response	Singing	Microphone	Commercial+built in-house	t_Pattern_: 0.15 s before or after the beat and up to 30% of the note duration Song duration: the note must be sung at least 60% of the time
Fruity Jump	Call and response	Tapping	Contact pressure	Built in-house	t_Perfect_: 0.2 s before or after the beat t_Good_: 0.25 s before or after the beat t_Correct_: 0.3 s before or after the beat
Karate Fruits	Last beat	Punching	Webcam	Built in-house	t_Perfect_: 0.08 s before or after the beat t_Good_: 0.14 s before or after the beat t_Correct_: 0.3 s before or after the beat

^a^Name of the task.

### Feasibility Study

We conducted a feasibility study to evaluate whether children with NDDs involving reading deficits could use Mila-Learn autonomously at home. Our main objective in assessing Mila-Learn’s autonomous use was to monitor both the time users spent on the SG and their accuracy in each game played. In the context of the unprecedented health crisis caused by COVID-19, participants were recruited by the French Federation for Learning Disorders (FFDys), a national association that aggregates all regional associations of people with learning disabilities. The FFDys communicated to its members the possibility of testing an app and managed the information and consent of participants. Families were informed that Mila-Learn was an SG for performing rhythmic tasks at home and that we believed this practice might be beneficial for learning to read. In total, 2500 children downloaded Mila-Learn. The analyses were conducted on a subsample of 21% (525/2500) of these children, who spontaneously played at least 15 games. To improve the usability of Mila-Learn, we also asked users (both children and families) to provide feedback on the games and information on the children’s impairments. This information was provided freely and was not compulsory to obtain Mila-Learn. In addition, we systematically collected through phone interviews all the problems that the children and their families encountered regarding the computing and web performance of the SG. Finally, we conducted a phone survey of 200 users, which is provided in Multimedia Appendix 1 [56]. The questions asked were designed to gain insights into the families’ perceptions of the benefits of the tool, the improvements and difficulties of use they encountered, and their desire to continue using the game in the future; in addition, room was left for unstructured testimony. The data analysis for the feasibility study was limited to descriptive statistics.

### Usability Study

This usability study was considered a continuation of study 1 and was conducted under the same ethical rules. In the usability study carried out in a real-life setting over 6 months, our primary focus was 2-fold following modifications to Mila-Learn based on study 1 feedback: first, to ensure that the computational architecture and final version of Mila-Learn were free of computer bugs and, second, to track player progress using Mila-Learn’s scoring system over an extended duration. As part of the second lockdown because of the COVID-19 crisis, the final version of Mila-Learn was made available again starting on October 10, 2020, on National Learning Disabilities Day. Benefiting from the large amount of feedback received during the first lockdown, very few technical problems occurred, resulting in a game with much better fluidity that provided higher-quality data. A total of 3337 children had access to Mila-Learn for a total of 84,682 games that were played. As in study 1, at the time of registration, the patients’ families were given the opportunity to complete the profile of the children, including information such as the children’s diagnoses. A total of 304 diagnoses were reported by the parents. Finally, the children and their families had the option of linking the game character to the reported clinical profile. This option was exercised by 2.94% (98/3337) of the children, for whom we had both their reported diagnosis and game performance over time. These 2.94% (98/3337) of the children completed 3922 games.

To assess how children performed with Mila-Learn, we defined and computed the following variables:

1. “Time” is an incremental value representing the number of levels played by a player since the beginning of the experiment. Time is 1 at the beginning of the experiment and represents the total number of levels played by the player at the end of the experiment.
2. “Delta_tap” is the delay between the date of the played input (as defined in Table 1) and the date of the expected input.
3. “Threshold” is a delay defined for each game that was used to construct the performance score.
4. “Performance score” is a variable bounded between 0 and 100 that was created to quantify performance from delta_tap and normalize performance across games. We used the following formulas: performance score = (–100/threshold) × abs(delta_tap) + 100 for abs(delta_tap)≤threshold and performance score=0 for abs(delta_tap)>threshold.

We conducted several linear mixed models. To assess children’s progress over time, we tested whether players improved their performance through the progression across the games using a linear mixed model with the following formula: performance score ~ time + (1|PlayerID/GameID/LevelName).

To assess whether a declared diagnosis was associated with the average performance of the children, we also conducted a linear mixed model using the following formula: performance score ~ age + dyscalculia + dysgraphia + dyslexia + dysphasia + ADHD + ExecutiveFunction impairment + (1|GameID/LevelName).

Finally, we also tested whether progress over time was moderated by a declared diagnosis using the following formula: performance score ~ time + diagnosis + time × diagnosis + (1|GameID/LevelName).

### Ethical Considerations

Under French legislation, we did not need the approval of a Comité de Protection des Personnes (Committee for the Protection of Persons). However, as the pilot study was conducted in line with the creation of large databases, we obtained the approval of the Commission Nationale de l’Informatique et des Libertés (National Commission for Informatics and Freedoms) under number 2222283.

## Results

### Feasibility Study

Between April 2020 and June 2020, a total of 2500 children had access to Mila-Learn. Families reported the child’s diagnosis in 60% (1500/2500) of cases. As children were recruited through the FFDys, they were diagnosed with an NDD in almost all cases, but only 23% (575/2500) were declared as having dyslexia. The other children had developmental coordination disorders (dyspraxia), dyscalculia, and communication disorders of oral language (dysphasia). In addition, 18% (450/2500) declared a diagnosis of ADHD.

Data regarding the use of Mila-Learn by each user were recorded as time spent on the SG and accuracy in each game played. The average use was 3.5 sessions per week. To ensure the significance of the data, we only kept the data of players who participated over a sufficient period (>15 games). Duration was expressed as the number of games played. We considered the number of games played inside the SG over the number of played sessions as the number of games played in 1 session could vary widely. In total, 21% (525/2500) of players aged 6 to 14 years played at least 15 games, with an overall mean of 54.77 and a median of 42 games played. The average number of games played was similar across ages (no main effect of age). No effect of age was found on the mean score. In addition, no floor or ceiling effects were observed (Multimedia Appendix 2).

It should be noted that several technical issues occurred during the first 2 weeks owing to the wide variety of tablet operating systems. This situation resulted in the deployment of corrective patches, but owing to the correction delay, it may have differentially altered one child’s experience relative to another’s. To improve the user experience, phone calls were systematically conducted to interview families, determine potential problem areas, and gather feedback for improvement. Parents consistently highlighted the recreational side of the game and its impact on the children’s self-confidence. A survey of 200 users, provided in Multimedia Appendix 1, also indicated that 96% (192/200) wished to continue using Mila-Learn after the COVID-19 pandemic. However, they also provided significant feedback (164/200, 82%) to improve the game. Multimedia Appendix 3 [57] presents the most significant feedback with a frequency of ≥10 occurrences. We classified it according to the criteria by Morville [56], which distinguish 7 dimensions: usefulness, usability, findability (the ease of locating a feature or a piece of context), credibility, accessibility design, attractiveness, and value [48]. Usability was questioned in several comments, such as “the detection of movements should be improved,” “sound detection needs to be improved,” and “the game needs to be better adapted to the child’s difficulty profile.” Accessibility was also questioned as several parents indicated that “the writing could benefit from being larger and the display of dialogues slower.”

### Mila-Learn Description Adjustments Following Study 1

#### Design Framework

On the basis of the feedback obtained during study 1, we made several modifications to Mila-Learn. To improve accessibility, the first modification was to offer the player the choice between several fonts, including OpenDyslexic. This choice is reversible throughout the game. We also improved sound and movement detection. A second significant choice was to distinguish the children’s pathways according to their predominant disorders to facilitate their entry into the game and usability. For example, a player who indicated that they had dyspraxia at the time of registration was offered more moderate motor exercises (ie, Sing Lab), allowing them to enter the adventure before training on River Splash or Karate Fruits that are more challenging in terms of motor abilities. In contrast, a child with dyslexia could be offered River Splash from the beginning, with Sing Lab exercises being offered only afterward as Sing Lab involves the phonological loop.

To increase motivation and interest in the game, we provided new possibilities of personalization for the character: the player could choose the gender of the avatar, their color, and the color of the hat. We then increased the storyline with the help of a screenwriter. This modification improved the consistency of the story and made it more inclusive by adding new characters that could help the player during the game. A new companion named “Mila” appeared, who is a fairy representing the planet “Mila” where the story takes place. These modifications also influenced (1) the dialogues, which were shortened with the language adapted to children; and (2) the appearance of the notion of “rhythmic,” which was introduced as a martial art based on rhythm to clarify the main goal of the game during the adventure.

Through this expansion, we created 6 new chapters. We maintained the same concept as the preceding version and gradually increased the level of difficulty during the progression of the game by increasing the speed of the rhythm and varying the type of rhythm clapped (ie, clapping notes, then eighth notes). We also created daily missions. These 4 daily tasks allowed the child to revisit games on which they had practiced in the past and where they encountered difficulties. This allowed us to directly address the tendency to forget what has been learned and allowed for longer practice with Mila-Learn.

#### Description of the Tasks

Finally, we made structural modifications to the proposed tasks to ensure the game’s fluidity and improve motor interactions. First, we changed the way children had to answer during Clap Trap. Instead of clapping both hands, which was recorded using the microphone, we changed the child’s interaction with the SG to synchronously tapping both hands on the screen (and, therefore, we used a touch recording). Second, Follow me was extensively modified to be more understandable and involve the child more on a motor level. The interaction was changed from a passive mode (one contact pressure) to a more active hand clapping measured using the microphone. The child did not perform the task all at once but interacted with the character, who gave them instructions that the child reproduced on the principle of call and response. Specific music was created for the game. As a consequence, Follow me was renamed Clap Hero. Finally, we modified the way children had to answer during Fruity Jump—children’s interaction with Mila-Learn changed from tapping to shaking the tablet, which was measured using an accelerometer. Table 2 summarizes the changes made in the final version of Mila-Learn.

**Table 2:** Players’ recorded responses and game parameters in the final version of Mila-Learn.

Task	Type	Interaction	Capture technology	Songs	Tolerance threshold
Clap Hero	*Call and response* ^a^	*Clapping hands*	*Microphone*	*Customized*	Perfect: 0.1 s before or after the beat Good: 0.15 s before or after the beat Correct: 0.25 s before or after the beat
Clap Trap	Last beat	*Tapping on the left and right side of the screen*	*Touch*	Commercial	Perfect: 0.1 s before or after the beat Good: 0.15 s before or after the beat Correct: 0.25 s before or after the beat
River Splash	Last beat	Shaking tablet	Accelerometer	Commercial	Perfect: 0.1 s before or after the beat Good: 0.15 s before or after the beat Correct: 0.25 s before or after the beat
Sing Lab	Call and response	Singing	Microphone	*Customized*	Pattern: 0.15 s before or after the beat and up to 30% of the note duration Song duration: the note must be sung at least 60% of the time
Fruity Jump	Call and response	*Shaking tablet*	*Accelerometer*	Customized	Perfect: 0.2 s before or after the beat Good: 0.25 s before or after the beat Correct: 0.3 s before or after the beat
Karate Fruits	Last beat	Punching	Webcam	Customized	Perfect: 0.08 s before or after the beat Good: 0.14 s before or after the beat Correct: 0.3 s before or after the beat

^a^Italics indicate game and functional changes that were introduced compared with the Mila-Learn second version summarized in Table 1.

### Usability Study

This usability study focused on a sample of 98 children (mean age 9.05; SE 2.4 years), and we had both their reported diagnoses and game performance over time. These 98 children completed 3922 games. The linear mixed models yielded the following significant results. First, we found that the performance of the children significantly improved over time (β=.02; *t*_3268_=2.68; *P*=.007). That is, there was an increase in the performance score by an average of 5 points after 250 levels were played.

Second, we explored whether declared diagnosis and age influenced the average performance of the children. Table 3 summarizes the results. We found that older children performed better than younger children. One year of age increased the normalized performance score by 1.08 points (meaning 1.1% of the maximal range). In addition, children with dyslexia and ADHD performed significantly better than those with other diagnoses (performance improved significantly faster in children with ADHD, β=.06; *t*_3754_=3.91; *P*<.001, and slower in children with dyslexia, β=−.06; *t*_3816_=–5.08; *P*<.001). Having dyslexia increased the normalized performance score by 2.81 points (meaning 2.8% of the maximal range) compared with children without dyslexia, whereas having ADHD increased the normalized performance score by 4.16 points (meaning 4.2% of the maximal range) compared with children without ADHD. In contrast, children with executive function impairment and dysgraphia performed significantly worse than those with other diagnoses. Having dysgraphia decreased the normalized performance score by 2.06 points (meaning 2.1% of the maximal range) compared with children without dysgraphia, whereas having executive function impairment decreased the normalized performance score by 3.26 points (meaning 3.3% of the maximal range) compared with children without executive function impairment.

Finally, we also tested whether progress over time statistically interacted with the declared diagnosis. We found that children with ADHD progressed faster over time than those with other diagnoses (β=.06; *t*_3754_=3.91; *P*<.001) and that children with dyslexia (β=−.06; *t*_3816_=–5.08; *P*<.001) and executive dysfunction (β=−.03; *t*_3805_=–2.09; *P*=.04) improved less over time than those with other diagnoses. We found no significant interaction between time and a diagnosis of dysphasia (β=−.01; *t*_3816_=–0.68; *P*=.50), dyscalculia (β=.05; *t*_3787_=1.46; *P*=.14), or dysgraphia (β=.00; *t*_3816_=–0.1; *P*=.92).

**Table 3:** Average scores according to diagnosis during study 3 with the final version of Mila-Learn.

	Estimate (SE)	*t* test (*df*)	*P*r(>|*t*|)
Intercept	37.83 (4.60)	8.23 (6.04)	<.001
Age	1.08 (0.15)	7.02 (2082.28)	<.001
Dyscalculia (yes)	−0.43 (1.03)	−0.42 (2081.02)	.68
Dysgraphia (yes)	−2.06 (0.79)	−2.60 (2084.61)	.009
Dyslexia (yes)	2.81 (0.70)	4.03 (2087.52)	<.001
Dysphasia (yes)	−0.51 (0.93)	−0.54 (2086.54)	.59
ADHD^a^ (yes)	4.16 (0.61)	6.79 (2090.71)	<.001
Executive function impairment (yes)	−3.26 (1.36)	−2.40 (2086.78)	.02

^a^ADHD: attention-deficit/hyperactivity disorder.

## Discussion

### Principal Findings

The literature on SGs, especially when designed for a specific medical condition, is limited when it focuses on game design methodology or formal clinical validation [42]. In this paper, we described the process and empirical studies to address this issue for Mila-Learn, an SG based on rhythmic training for children with dyslexia. To do so, we placed the patient’s experience at the center of the game construction while iterating with clinicians involved in treating children with dyslexia. In this paper, we described the different developmental phases that helped us design the game. We first constructed an initial prototype based on a literature review and with the help of clinicians specializing in learning disorders. Then, based on a first round of feedback from users and comments from professionals, we developed a first version of Mila-Learn for tablets.

In this version, we greatly improved the users’ experience with the game by adding new gaming features to increase the motivation and engagement of players. We offered more possibilities for customization, created a storyline, and introduced humorous and friendly characters to align with children’s interests [51,52]. Moreover, we adapted the difficulty of the game to enhance the learning possibilities of children by working on graphism and the instructions given to the children and by creating evolving tasks that gradually increased the level of difficulty [51,53,54]. With this second version, we adopted a user participatory design by inviting children, families, and professionals to test this version and send us feedback about their experience (feasibility study). User participatory design is a method that is currently gaining attention. Contrary to user-centered designs, which create games for a user, participatory design aims to construct the game with the users by collecting their experience and advice and then including them in the game [58]. It has been shown that participatory design promotes engagement of the user [52]. Indeed, collecting feedback both from families and children and from professionals is essential as professionals and families and children focus on different aspects of an SG and do not place the same importance on each feature [52]. We believe that this participative process helped us develop an SG that improved the experience within the game and the interest of families and children in Mila-Learn.

Regarding computational aspects, we also collected feedback that helped us resolve bugs and record the time spent on the game and the player’s accuracy in each game. These features allowed us to follow children’s interest in the game and their progression over time and demonstrate that progression occurred with Mila-Learn and was associated with age. Study 1 confirmed that children could engage with Mila-Learn for a rather long period and play at home without the need for an extra supporting person, suggesting that Mila-Learn was sufficiently motivating and adapted to this population. Children and their families appeared to be highly satisfied with the game.

Finally, following a third round of feedback from parents, children, and professionals, we developed a final version of Mila-Learn to improve accessibility and motivation for the player. We made structural modifications to the proposed tasks to ensure the fluidity of the game and improve motor interactions. We resolved most of the technical problems, which allowed us to conduct a real-life usability study of the Mila-Learn game during the second lockdown.

### Comparison With Prior Work

In the usability study, we observed that children significantly improved their scores on the 6 games included in Mila-Learn. Although we cannot conclude that the rhythm abilities of the children improved based only on these results, we believe that the children learned how to use Mila-Learn and that they were increasingly accurate in responding to each game. However, the effect size was small, although it may have been underestimated as the difficulty in the games increased, which could have masked the children’s progression. In addition, based on the diagnosis declared by the children’s parents, we performed exploratory analyses to assess whether improvements over time were associated with the declared diagnoses. Linear mixed models showed that children’s performance significantly increased over time, that scores were better for children with ADHD and dyslexia, and that performance improved significantly faster for children with ADHD and slower for children with dyslexia.

Regarding the average performance of children according to diagnosis, the results were very encouraging if we consider the relationship between reading impairments and diagnosis. On the basis of the literature, we expected reading impairments to be associated with dyslexia, attention deficit, and specific oral language impairment (dysphasia) [59,60]. In addition, we expected that severity would negatively influence performance. This is usually the case when children have dysphasia [61], executive function impairment [62], or multidimensional impairments [63]. The results were in line with these expectations. Children with dyslexia and ADHD showed a significantly better performance over time, whereas children with dysphasia, executive function impairment, and dysgraphia showed a worse performance. Of note, children with dysgraphia often have motor coordination disorders [64]. Finally, dyscalculia had no influence on Mila-Learn performance. In summary, the predictions according to diagnosis were in line with the hypothesis that Mila-Learn may improve performance in children with reading impairments. The fact that dyscalculia showed no specific effects and that the diagnoses associated with the highest severity (dysphasia and executive function impairment) showed less improvement followed our hypothesis [61-63,65]. We speculated that dysgraphia was associated with multidimensional impairments, including motor coordination disorder. This interpretation is based on the fact that recruitment for the study was based only on user reading impairments.

Regarding the average performance of children according to diagnosis over time (ie, the statistical interaction), the fact that performance improved significantly faster for children with ADHD and slower for children with dyslexia is not surprising as the perception of rhythm is impaired in children with dyslexia. In contrast, children with ADHD may have impairments in reading abilities but do not have specific deficits in rhythm and speech perception [57,66].

### Strengths and Limitations

The exploratory studies presented in this paper have some limitations despite the promising results. On the one hand, some aspects of the game need to be improved.

First, we currently consider a “standard” latency of 20 ms, which corresponds to the estimated delay between the child’s real input and the input processed by the operating system. In reality, each tablet may have unique differences. In the next version of Mila-Learn, we need to consider this unique latency to get as close as possible to the real performance of children. This adjustment might lead to more accurate measurements of children’s interactions and, potentially, more tailored game experiences.

Second, the game gradually increases in difficulty with the progression of the player within the game. We integrated some specific pathways as a function of the difficulties that the children declared before starting the game (ie, children with motor difficulties do not start with games that require a high level of motor skills). However, the progression is predetermined and does not take into account the results of the player. In the next version of Mila-Learn, the difficulty of each game will automatically adapt based on the child’s performance in the previous games using a specific algorithm [53], allowing for much better stimulation. By doing so, the game could offer a more individualized experience, potentially leading to more sustained engagement and greater benefits for the children.

Third, the age range of 7 to 14 years is wide as children’s interests can vary greatly during these years. In a future version of Mila-Learn, the graphics and music will be adapted to the age specified by the child so that the game will be more suitable for their age. This may enhance the game’s appeal to players across the entire age range, fostering increased engagement and learning.

However, our study was only exploratory in nature. First, even if the lockdown gave us the opportunity to have a large sample for exploratory studies, diagnoses were not clinically grounded and were only declared by the children’s parents. Therefore, caution should be the rule when interpreting predictive models.

Second, there was no predefined design for the studies as the training was spontaneous and included no comparison with alternative treatment proposals. Therefore, the clinical interest of Mila-Learn for dyslexia cannot be established based on the results of the 2 exploratory studies presented in this paper.

### Future Directions

To address the clinical relevance of Mila-Learn in relation to dyslexia, the next step will be to evaluate the effects of Mila-Learn in the context of a randomized controlled trial. Children with dyslexia based on objective clinical assessments will be randomized to Mila-Learn sessions or placebo game sessions that take place in the same universe but with different tasks. We will assess the evolution of reading skills from before to after training with the hope of greater improvements with Mila-Learn. On the basis of the exploratory studies, we calculated the number of patients per group that would ensure a statistical power of at least 85% for an effect size equal to 0.5 (moderate) when the changes in the experimental and control groups were compared. This calculation indicated that each group should have at least 73 children (ie, 146 children in total). This study started in September 2021 (Comité de Protection des Personnes registration 2021-A01709-32; ClinicalTrials.gov Identifier: NCT05154721).

### Conclusions

We presented how we constructed Mila-Learn, an SG based on rhythm activities, to improve reading skills in children with dyslexia. We developed several versions of the game considering the literature, professionals’ experiences, and users’ feedback. We also conducted a usability and a feasibility study to evaluate each version of Mila-Learn. The results indicated that Mila-Learn was attractive and sustained the players’ motivation and engagement for several months. Moreover, children were able to learn how to use the game, and their performance in the games improved with training. Future research will include (1) adapting to the latency of the electronic devices, (2) automatically adapting the games based on the player’s performance, and (3) conducting a large randomized controlled trial to evaluate the impact of Mila-Learn on reading skills.

## Appendix

Multimedia Appendix 1 User survey on Mila-Learn.

Multimedia Appendix 2 Average scores from all games between April 2020 and June 2020 according to the children’s age.

Multimedia Appendix 3 Feedback classification based on the criteria by Morville [55].

## Abbreviations

ADHD: attention-deficit/hyperactivity disorder
FFDys: French Federation for Learning Disorders
NDD: neurodevelopmental disorder
SG: serious game

## Data Availability

The data sets generated during and/or analyzed during this study are available from the corresponding author on reasonable request.
