# Supplementary material for: A Serious Game to Train Rhythmic Abilities in Children With Dyslexia: Feasibility and Usability Study
Source: JMIR Serious Games. 2024 Jan 11;12:e42733. doi: 10.2196/42733 (PMC10811594; doi:10.2196/42733)
Supplement: Multimedia Appendix 1 [file games_v12i1e42733_app1.docx]

**Appendix 1 : User Survey on MILA-Learn**

**This appendix provides the user survey designed to gather feedback from users regarding their experience with MILA. Participants had the freedom to answer the questions in an open-ended format and were not obligated to provide a response to every question. The responses to these questions aim to inform future improvements and adjustments to the system.**

**Survey Questions**

1. **Primary Utility of MILA
   *Quelle est la principale utilité que vous tirez de Mila?*
   [Open response]**
2. **Technical Improvements and Issues
   *Quelles sont les améliorations et avancées techniques à mettre en oeuvre? Vous pouvez nous renseigner aussi les problèmes techniques rencontrés.*
   [Open response]**
3. **Additional Feedback
   *Avez-vous d'autres témoignages à nous communiquer?*
   [Open response]**
4. **Future Use
   *Souhaitez-vous poursuivre l'utilisation de Mila à l'avenir?*
   [Open response]**
