# Supplementary figures and images for: A Serious Game to Train Rhythmic Abilities in Children With Dyslexia: Feasibility and Usability Study
Source: JMIR Serious Games. 2024 Jan 11;12:e42733. doi: 10.2196/42733 (PMC10811594; doi:10.2196/42733)

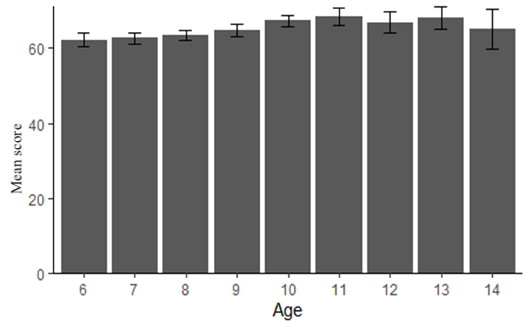

Supplement: Multimedia Appendix 2 [file games_v12i1e42733_app2.png]
