# Supplementary material for: A Serious Game to Train Rhythmic Abilities in Children With Dyslexia: Feasibility and Usability Study
Source: JMIR Serious Games. 2024 Jan 11;12:e42733. doi: 10.2196/42733 (PMC10811594; doi:10.2196/42733)
Supplement: Multimedia Appendix 3 [file games_v12i1e42733_app3.docx]

**Appendix 2 : Feedback Classification Based on Morville's Criteria.**

| **Occurence** | **Targeted quality of the device [66]** | **Quote example** |
| --- | --- | --- |
| 43 | Desirable | My son participates with pleasure, sometimes finds it too short, he wants more! What a pleasure to learn while having fun! |
| 26 | Valuable | The game helps my child concentrate and trains his coordination skills |
| 22 | Usable | The detection of movements should be improved |
| 14 | Desirable | The songs and music are well adapted |
| 12 | Usable | Sound detection needs to be improved |
| 11 | Usable | The game needs to be better adapted to the child's difficulty profile |
| 10 | Accessible | The writing could benefit from being larger and the display of dialogues slower |
| 10 | Useful | It is a great complement to rehabilitation |
